# Isolation and Identification of a Rare Spike Gene Double-Deletion SARS-CoV-2 Variant From the Patient With High Cycle Threshold Value

**DOI:** 10.3389/fmed.2021.822633

**Published:** 2022-01-06

**Authors:** Li-Teh Liu, Jih-Jin Tsai, Chun-Hong Chen, Ping-Chang Lin, Ching-Yi Tsai, Yan-Yi Tsai, Miao-Chen Hsu, Wan-Long Chuang, Jer-Ming Chang, Shang-Jyh Hwang, Inn-Wen Chong

**Affiliations:** ^1^Department of Medical Laboratory Science and Biotechnology, College of Medical Technology, Chung-Hwa University of Medical Technology, Tainan, Taiwan; ^2^Tropical Medicine Center, Kaohsiung Medical University Hospital, Kaohsiung, Taiwan; ^3^Division of Infectious Diseases, Department of Internal Medicine, Kaohsiung Medical University Hospital, Kaohsiung, Taiwan; ^4^School of Medicine, College of Medicine, Kaohsiung Medical University, Kaohsiung, Taiwan; ^5^National Mosquito-Borne Diseases Control Research Center, National Health Research Institutes, Zhunan, Taiwan; ^6^National Institute of Infectious Diseases and Vaccinology, National Health Research Institutes, Zhunan, Taiwan; ^7^Division of Hepatobiliary and Pancreatic, Kaohsiung Medical University Hospital, Kaohsiung, Taiwan; ^8^Division of Nephrology, Department of Internal Medicine, Kaohsiung Medical University Hospital, Kaohsiung, Taiwan; ^9^Department of Medical Research, Kaohsiung Medical University Hospital, Kaohsiung, Taiwan; ^10^Department of Internal Medicine and Graduate Institute of Medicine, Kaohsiung Medical University, Kaohsiung, Taiwan; ^11^Department of Pulmonary Medicine, Kaohsiung Medical University Hospital, Kaohsiung, Taiwan

**Keywords:** COVID-19, SARS-CoV-2, RT–PCR, Ct, virus culture, spike gene, variant, phylogenetic analysis

## Abstract

Coronavirus disease 2019 (COVID-19) is an emerging life-threatening pulmonary disease caused by infection with severe acute respiratory syndrome coronavirus 2 (SARS-CoV-2), which originated in Wuhan, Hubei Province, China, in December 2019. COVID-19 develops after close contact via inhalation of respiratory droplets containing SARS-CoV-2 during talking, coughing, or sneezing by asymptomatic, presymptomatic, and symptomatic carriers. This virus evolved over time, and numerous genetic variants have been reported to have increased disease severity, mortality, and transmissibility. Variants have also developed resistance to antivirals and vaccination and can escape the immune response of humans. Reverse transcription polymerase chain reaction (RT–PCR) is the method of choice among diagnostic techniques, including nucleic acid amplification tests (NAATs), serological tests, and diagnostic imaging, such as computed tomography (CT). The limitation of RT–PCR is that it cannot distinguish fragmented RNA genomes from live transmissible viruses. Thus, SARS-CoV-2 isolation by using cell culture has been developed and makes important contributions in the field of diagnosis, development of antivirals, vaccines, and SARS-CoV-2 virology research. In this research, two SARS-CoV-2 strains were isolated from four RT–PCR-positive nasopharyngeal swabs using VERO E6 cell culture. One isolate was cultured successfully with a blind passage on day 3 post inoculation from a swab with a Ct > 35, while the cells did not develop cytopathic effects without a blind passage until day 14 post inoculation. Our results indicated that infectious SARS-CoV-2 virus particles existed, even with a Ct > 35. Cultivable viruses could provide additional consideration for releasing the patient from quarantine. The results of the whole genome sequencing and bioinformatic analysis suggested that these two isolates contain a spike 68-76del+spike 675-679del double-deletion variation. The double deletion was confirmed by amplification of the regions spanning the spike gene deletion using Sanger sequencing. Phylogenetic analysis revealed that this double-deletion variant was rare (one per million in public databases, including GenBank and GISAID). The impact of this double deletion in the spike gene on the SARS-CoV-2 virus itself as well as on cultured cells and/or humans remains to be further elucidated.

## Introduction

A novel coronavirus, named 2019-nCoV, was isolated from lower respiratory tract samples collected from patients with pneumonia, and this virus was identified in Wuhan, Hubei Province, China on December 21, 2019 (1). The disease caused by 2019-nCoV was named COVID-19, which is short for coronavirus disease 2019 and was named by the World Health Organization (2). The virus was later renamed severe acute respiratory syndrome coronavirus 2 (SARS-CoV-2) (3). This is the seventh human-infecting coronavirus (1, 4–7) and is one of a few life-threatening coronaviruses (1, 4, 7), which belongs to the Sarbecovirus subgenus, the Betacoronavirus genus, the Orthocoronavirinae subfamily, the Coronaviridae family, Cornidovirineae suborder, and the Nidovirales order (3). The COVID-19 pandemic has resulted in 251,788,329 cumulative cases and 5,077,907 deaths worldwide as of November 12, 2021 (https://covid19.who.int/); these numbers increase daily. Although the results of phylogenetic analyses have suggested that SARS-CoV-2 is closely related to SARS-like betacoronaviruses of bat origin, the genomic structure of SARS-CoV-2 is more similar to that of SARS-CoV (8). SARS-CoV-2 is an enveloped RNA virus consisting of a single-stranded positive-sense RNA genome of ~30 kilobases. The genome is composed of 11 coding regions that encode 12 potential gene products, including ORF1a, ORF1b, Spike (S), ORF3a, Envelope (E), Membrane (M), ORF6, ORF7a, ORF7b, ORF8, Nucleocapsid (N), and ORF10 (9). SARS-CoV-2 spreads widely and spreads primarily via active pharyngeal viral shedding and respiratory droplets by asymptomatic, presymptomatic, and symptomatic carriers during close contact, including talking, coughing, or sneezing (10). Although the evolution rate of SARS-CoV-2 is slower than that of other RNA viruses (e.g., HIV-1 or influenza virus), due to the genetic proofreading mechanisms among coronaviruses (11, 12), natural selection can work on rare but beneficial mutations. The dark side of virus evolution to humans is that genetic variations result in an increase in disease severity, mortality, transmissibility, resistance to antivirals and vaccination and escape from the immune response of humans. Accumulating SARS-CoV-2 variants are categorized as variants of interest (VOIs), variants of concern (VOCs), and variants under monitoring (VUMs). To date, five SARS-CoV-2 lineages, namely, alpha, beta, gamma, delta, and omicron variants, have been designated VOCs that have increased transmissibility and disease severities (13). SARS-CoV-2 has been detected in different types of clinical specimens, including bronchoalveolar lavage fluid, sputum, nasal swabs, pharyngeal swabs, feces, blood, and urine (14, 15). The entry of this virus is mediated by the binding of the cellular entry receptor angiotensin-converting enzyme 2 (ACE2), cofactors such as cellular serine protease TMPRSS2 (16) and potentiating factor neuropilin-1 (NRP1) (17).

Alongside the common clinical symptoms of COVID-19, including fever, dry cough, and shortness of breath, the diagnosis of COVID-19 has been rigorously developed in the past 2 years. The methods for COVID-19 diagnosis are nucleic acid amplification tests (NAATs), serology tests, and diagnostic imaging, such as computed tomography (CT) (18–21). Reverse transcription polymerase chain reaction (RT–PCR) is the method of choice among these diagnostic techniques to detect COVID-19 at an early stage and is very useful in the diagnosis and prevention of COVID-19 (22). It has been suggested that the viral load determined by using RT–PCR is associated with increased disease severity and mortality (23–25). However, RT–PCR cannot distinguish genomic RNA fragments from transmissible live viruses in clinical specimens and could lead to false-negative results in the diagnosis of COVID-19 (15, 21). The isolation of SARS-CoV-2 by using cell culture from clinical samples was suggested as a surrogate marker for infectivity and helps to determine deisolation protocols (26). In addition, SARS-CoV-2 culture might play an important role in the development of antivirals, vaccines, and SARS-CoV-2 virology research.

In this study, we report the isolation and identification of a rare spike gene double-deletion SARS-CoV-2 variant from a patient with a high cycle threshold value (Ct) by VERO E6 cell culture with blind passage. Strategies for higher virus isolation rates were discussed, and the evolutionary relationship between the rare double-deletion variant and SARS-CoV-2 deposited in public databases was analyzed.

## Materials and Methods

### Sample Collection and Ethics Statement

The Tropical Medicine Center (TMC) with the facility of a biosafety level-3 laboratory of Kaohsiung Medical University Hospital (KMUH) obtained authority to perform SARS-CoV-2 culture from the Central Epidemic Command Center (CECC), Taiwan, in May 2020. The methods of SARS-CoV-2 qRT–PCR and virus culture were set up thereafter, and the investigation was performed in accordance with the laboratory biosafety guidelines of the Taiwan Centers for Disease Control (27). Nasopharyngeal swabs of suspected COVID-19 cases were collected in Universal Transport Medium (UTM) (Viral Transport Medium w/Special Swab was purchased from Creative Life Science Co. Ltd., New Taipei City, Taiwan) and were immediately sent for SARS-CoV-2 qRT–PCR. The swabs with positive results were subjected to virus culture on the same day or on the next day. The study was reviewed and approved by the Institutional Review Board of KMUH [KMUHIRB-E(I)-20200013].

### RNA Extraction and the SARS-CoV-2 qRT–PCR

For the detection of the SARS-CoV-2 RNA genome, RNA was extracted from 140 μL nasopharyngeal swab samples in UTM using the QIAamp Virus RNA mini kit (QIAGEN, Germany) by following the manufacturer's procedure. Five microliters of RNA was immediately subjected to SARS-CoV-2 one-step qRT–PCR using the LightCycler Multiplex RNA Virus Master kit (Roche Diagnostics, Mannheim, Germany) in an Mx3000P PCR System (Agilent, USA). The SARS-CoV-2-specific primers and probes for the RdRP, E and N genes for qRT–PCR are outlined in Supplementary Table 1 (28). A Ct value <40 was considered a positive result (29). RNAse-free water and the RNA extracted from hCoV-19/Taiwan/4/2020 (EPI_ISL_411927, an isolate obtained from Taiwan Centers for Disease Control) cell culture supernatant were used as a negative control and positive control, respectively, in the qRT–PCR in the study.

### SARS-CoV-2 Culture

The samples with positive qRT–PCR results were inoculated into VERO E6 cells, which were established from African green monkey kidney epithelial cells. VERO E6 cells were routinely maintained in Dulbecco's modified Eagle's medium (Thermo Fisher Scientific, Waltham, MA, USA) supplemented with 10% fetal bovine serum (Thermo Fisher Scientific, Waltham, MA, USA), 1x antibiotic-antimycotic (Thermo Fisher Scientific, Waltham, MA, USA) and 1 mM sodium pyruvate (Thermo Fisher Scientific, Waltham, MA, USA) at 37°C in 5% carbon dioxide. One hundred microliters of original UTM sample and sample diluted 2X and 4X with serum-free DMEM containing 2X antibiotic-antimycotic was inoculated into VERO E6 cells that were preseeded in a 24-well plate (1 × 10^5^ cells/well) in duplicate followed by incubation at 37°C and 5% CO_2_ for 1 h. Six hundred microliters of DMEM with 2% FBS was added to the well, the cells were maintained at 37°C in a 5% CO_2_ incubator, and the cytopathic effect (CPE) was observed daily. Basically, the CPE was confirmed by SARS-CoV-2 qRT–PCR of the culture supernatant. For the sample in which the CPE was not observed 3 days after inoculation, 100 μL of the culture supernatant was blindly passed into another well with a monolayer of VERO E6 cells. The cells were observed for CPE daily for up to 21 days. SARS-CoV-2 propagation was confirmed by qRT–PCR.

### Virus Titer Quantification Assays

The median tissue culture infectious dose (TCID_50_) was determined in a 96-well plate with VERO E6 cells at a density of 1X10^4^ cells per well. In brief, the supernatant collected from VERO E6 cells with CPE in SARS-CoV-2 culture was serially diluted with DMEM containing 2% FBS. The diluted virus solution (1 × 10^−1^ to 1 × 10^−10^) was inoculated into wells A to H in rows 1 to 10 in a 96-well plate with VERO E6 cells. The negative control was VERO E6 cells without any virus added, and these cells were placed in rows 11 and 12. On the day when CPE was observed, the cells were fixed with 4% formaldehyde and carefully washed twice with PBS. The cells were then stained with crystal violet (Tonyar Biotech. Inc., Taoyuan City, Taiwan), and the TCID_50_ was determined using the Reed and Muench method (30).

### RNA Library Construction and Whole Genome Sequencing

RNA was extracted from 140 μL of nasopharyngeal swab samples in UTM using a QIAamp Virus RNA mini kit (QIAGEN, Germany). Before constructing the RNA library, the virus copy number was determined by using a COVID-19 Multiplex 1-Step RTqPCR Kit (Topgen Biotechnology Co., Kaohsiung, Taiwan). A total of 10^9^ copies of SARS-CoV-2 genomic RNA were subjected to construction of the RNA library with the VAHTS Universal V8 RNA-seq Library Prep Kit for Illumina (Vazyme Biotech Co., Nanjing, China). Briefly, the RNA was fragmented into small pieces of ~150~200 nucleotides using divalent cations at 94°C for 8 min. Then, the fragmented RNA was reverse-transcribed to create the final cDNA library in accordance with the protocol provided by the manufacturer, and the average insert size for the paired-end libraries was 150 bp. After ligation with a barcode sequencing adapter, the qualified library (~300 bp) was further analyzed on a MultiNA MCE-202 (SHIMADZU Co., Kyoto, Japan) with a DNA 2500 Kit (SHIMADZU Co., Kyoto, Japan), and then we performed paired-end sequencing on a NovaSeq 6000 Sequencing System (Illumina, Inc., San Diego, USA) following the vendor's recommended protocol. A total of 20 million paired-end reads of 150 bp length were generated per sample using the Illumina (Illumina, Inc., San Diego, USA) paired-end RNA-seq approach. The paired-end reads were then trimmed for adapter sequences and filtered by a quality value (QV) ≥20 using fastp (v 0.19.5) (31), and read lengths of ≥145 bp were filtered by Filter FASTQ (v1.1.5) (32). The retained paired-end reads were merged using fastq-join (Version 1.1.2) (33). After that, all of the reads were assembled by Unicycler (v0.4.8.0) (34) into contigs.

### Validation of the Variants by PCR Amplification and Sanger Sequencing

The RNA was reverse-transcribed by using 4x VirDect 1-step RT–qPCR Master Mix with random primers (Topgen Biotechnology Co., Kaohsiung, Taiwan) to create cDNA. To enable a fast-sequencing approach, amplifications were performed using 10 ng cDNA with TopPLUS PCR Master Mix (Topgen Biotechnology Co., Kaohsiung, Taiwan) and specific target primer pairs (Supplementary Table 1) with a working concentration of 250 nM on an Applied Biosystems 9700 Thermal Cycler (Applied Biosystems, USA) according to the manufacturer's instructions. The thermal cycling program used a protocol of 95°C for 3 min, 32 cycles of 95°C for 15 s, 60°C 20 s 72°C for 40 s, and a final extension of 72°C for 2 min. The amplified products were purified with VAHTS DNA Clean Beads (Vazyme Biotech Co., Nanjing, China) and were analyzed on the MultiNA MCE 202 with DNA 2500 Kit (SHIMADZU Co., Kyoto, Japan) to check the target amplicon length and quantity, and then the amplified products were used for Sanger sequencing according to manufacturer's protocol to confirm the single nucleotide variations (SNVs) and indel regions, respectively.

### The SARS-CoV-2 Genomes and Evolutionary Analysis

The SARS-CoV-2 genomes were retrieved from the GISAID EpiCoV (https://www.gisaid.org/) and NCBI GenBank (https://www.ncbi.nlm.nih.gov/genbank/) databases. The selected SARS-CoV-2 sequences were aligned by MAFFT 7.490 (https://mafft.cbrc.jp/alignment/software/) (35). The evolutionary history was inferred by using ModelFinder to find the most appropriate evolutionary model (36), and a theoretical phylogenetic tree was reconstructed (bootstrap replication number 1000) using IQ-TREE 2.1.3 COVID-edition (37).

## Results

### Detection and Isolation of SARS-CoV-2 in Nasopharyngeal Swab Samples

The COVID-19 case numbers in Taiwan were relatively low compared to those in most countries in the world during 2020 (Supplementary Figure 1). KMUH is located in Kaohsiung city, where COVID-19 is rarely found within Taiwan (Supplementary Figure 2). We collected nasopharyngeal swabs from four suspected COVID-19 patients who had just returned from traveling abroad and who had upper respiratory tract syndromes, and these patients were assigned by the CECC to KMUH for diagnosis and treatment from October to November 2020. SARS-CoV-2 genomic RNA was detected in the four nasopharyngeal swab samples using qRT–PCR as described in the Methods and Materials. Of these four samples, SARS-CoV-2 was isolated from two of the nasopharyngeal swabs by using VERO E6 cell culture. The Ct values of sample number 4 were 35.93, 36.26, and 36.98 for the E, RdRP and N genes, respectively, which were relatively high compared to those of sample number 1 (Table 1). SARS-CoV-2-induced CPE was observed, and the presence of SARS-CoV-2 genomic RNA was detected by using qRT–PCR (Table 1). It is worth noting that a CPE was not observed 3 days post-inoculation for sample number 4 using the original nasopharyngeal swab-UTM, while a CPE was observed 14 days post-inoculation with blind passage on day 3, which is the day that a CPE was not observed under microscope examination for sample number 4 (Supplementary Figure 3). CPEs were not observed for sample numbers 2 and 3 21 days post-inoculation, even though we did a blind passage on day 3 post inoculation. The two SARS-CoV-2 isolates were named KMUH-1, which was isolated from the sample of a traveler returned from the United Arab Emirates, and KMUH-2, which was isolated from the sample of a traveler from the Philippines. Neither patient received specific therapy because they had self-limiting and mild symptoms.

**Table 1:** Detection of the presence of SARS-CoV-2 using qRT–PCR and CPE on VERO E6 cells.

	**Nasopharyngel swab**^**a**^ **(Ct)**			**CPE observed DPI** ^**b**^	**Culture fluid from cells with CPE**^**c**^ **(Ct)**				
**Sample number**	**E gene**	**RdRP gene**	**N gene**		**E gene**	**RdRP gene**	**N gene**	**Isolate**	**TCID_50_^g^**
1	15.78	18.97	20.95	3^d^	13.72	17.45	23.52	KMUH-1	10^4.8^
2	36.96	35.93	37.66	-	ND^f^	ND	ND	-	-
3	36.27	36.45	38.63	-	ND	ND	ND	-	-
4	35.93	36.26	36.98	14^e^	11.85	16.15	19.73	KMUH-2	10^5.5^

a *RNA extracted from nasopharyngeal swab-UTM*.

b *DPI: days post-inoculation*.

c *RNA extracted from the culture fluid from cells with CPEs*.

d *CPEs were observed in cells inoculated with original nasopharyngeal swab-UTM on day 3*.

e *CPEs were observed only in cells with blind passage on day 14*.

f *ND, not determined*.

g *TCID_50_, Median Tissue Culture Infectious Dose*.

### Analysis of the KMUH-1 and KMUH-2 WGS Data

**Figure 1:**
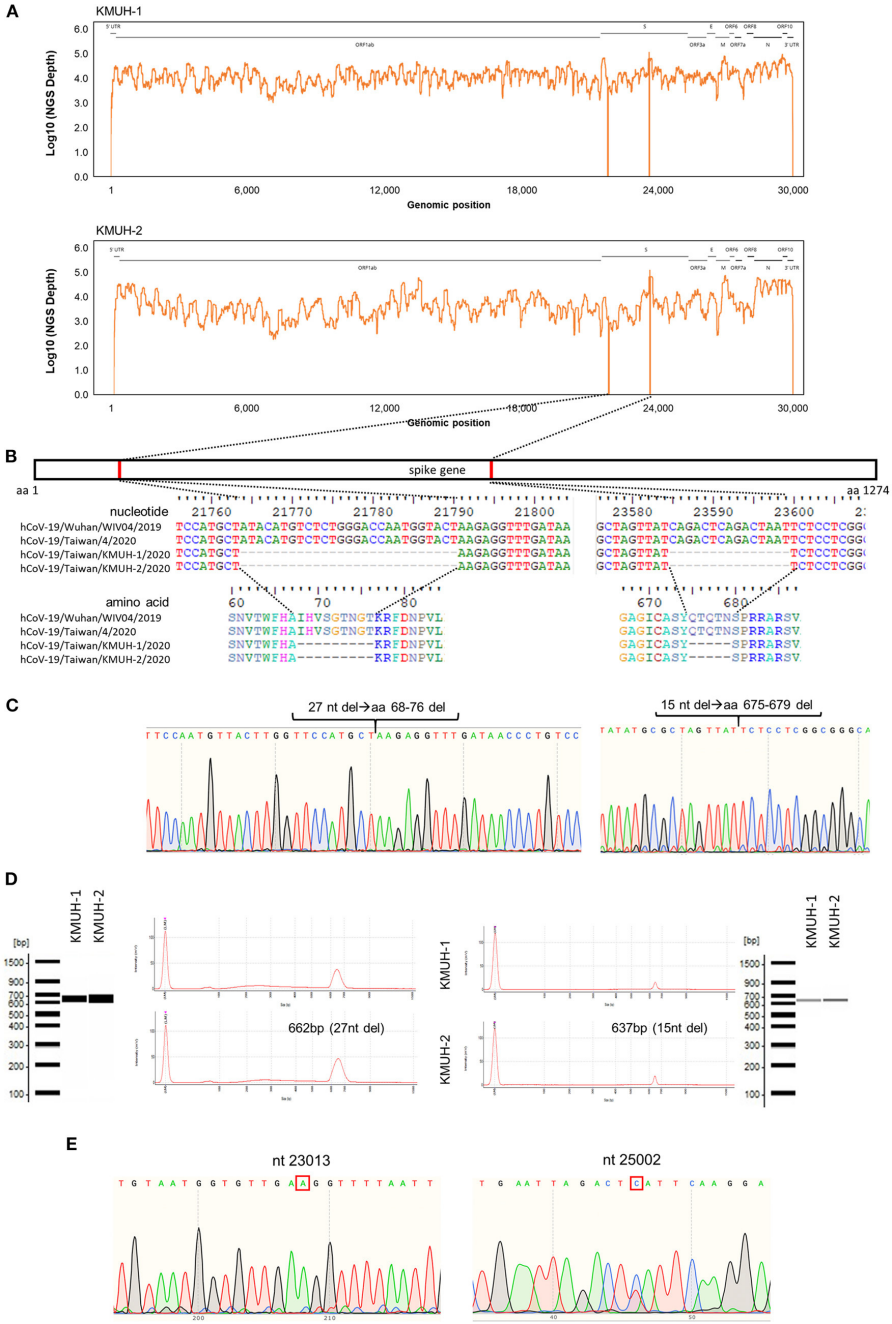
In-frame deletion and SNV in the spike gene in the SARS-CoV-2 genome. **(A)** WGS coverage and depths of KMUH-1 and KMUH-2. **(B)** Genomic regions of the spike gene deletion according to the genomic positions of the reference strain Wuhan-Hu-1/2019. **(C)** Results of Sanger sequencing of the regions spanning deletions in the spike gene in KMUH-1 and KMUH-2. **(D)** Deletions in the spike gene were verified by RT–PCR, which showed a reduced amplicon size. **(E)** Sanger sequencing of nt positions 23,014 and 25,002, which resulted in E484D and S1147 L aa substitutions in the spike protein. The Sanger sequencing results of KMUH-1 were used as a representative.

Complete genomic RNA sequences and data of the two SARS-CoV-2 isolates were uploaded to the GISAID EpiCoV database (https://www.gisaid.org/), and the amino acid substitutions were automatically reported in detail when compared to the reference, which was hCoV-19/Wuhan/WIV04/2019 (EPI_ISL_402124). The WGS coverage and depth distribution of KMUH-1 (EPI_ISL_5263327 and GenBank OL739246) and KMUH-2 (EPI_ISL_5395635 and GenBank OL739269) are shown in Figure 1A, and they were analyzed using Wuhan/WIV04/2019 as a reference sequence. Alignment and sequence comparisons of KMUH-1 and KMUH-2 to the reference sequence NC_045512.2 The SARS-CoV-2 isolate Wuhan-Hu-1/2019 was performed using Nextclade v1.7.3 (https://clades.nextstrain.org) (38) to verify the results of the virus detail report from GISAID. The results revealed two deletions, a 27-nucleotide (nt) and a 15-nt sequence at positions 21,764–21,790 and 23,585–23,599, in the spike genes of both KMUH-1 and KMUH-2, as shown in Figure 1B, Table 2. This double deletion resulted in amino acid (aa) 68-76 deletion and aa 675-679 deletion in the spike protein predicted *in silico*. The double-deletion variation was confirmed by using RT–PCR to check the target amplicon length (Figure 1C) and by Sanger sequencing (Figure 1D) using clinical samples to rule out possible mutation events during virus culture. In addition to the double deletion, the results suggested that there were SNVs in the orf1ab, spike and n genes (Table 2). The SNVs at nt positions 23,014 and 25,002, which resulted in E484D and S1147 L aa substitutions in the spike protein, were confirmed by RT–PCR and Sanger sequencing (Figure 1E). These double deletions and SNVs together resulted in coverages of 99.85% and 99.84% and depths between 2000 and 60000 in KMUH-1 and KMUH-2, respectively, when compared to the reference sequence.

**Table 2:** The sequence variation of KMUH-1 and KMUH-2 compared to the reference Wuhan-Hu-1/2019.

**Isolate**	**Position**	**Gene**	**Reference^**a**^**	**Variant**	**Affected codons**
KMUH-1	334	ORF1ab	T	C	V23 V
	3466	ORF1ab	T	C	H2067H
	10132	ORF1ab	A	G	T3289T
	19170	ORF1ab	C	T	S6302 L
	21764	Spike	GCTATACATGTCTCTGGGACCAATGGTA	G	68-76del
	23585	Spike	TATCAGACTCAGACTA	T	675-679del
	25002	Spike	C	T	S1147 L
	28887	N	C	T	T205I
	29864	—	A	C	—
KMUH-2	10132	ORF1ab	A	G	T3289T
	19169	ORF1ab	C	T	S6302 L
	21764	Spike	GCTATACATGTCTCTGGGACCAATGGTA	G	68-76del
	23014	Spike	A	C	E484D
	23341	Spike	T	C	G539G
	23585	Spike	TATCAGACTCAGACTA	T	675-679del
	25002	Spike	C	T	S1147 L
	28887	N	C	T	T205I

a *The nucleotides highlighted were deleted in KMUH-1 and KMUH-2 compared to the reference sequence*.

### Model Selection and Phylogenetic Tree Construction

To understand the evolutionary relationships of the two isolates in this study with similar strains, we downloaded the appropriate SARS-COV-2 genomes from the GISAID and GenBank databases. First, we searched highly similar SARS-COV-2 variants by sequence mining using Betacoronavirus BLAST (BLASTN) (39) using the KMUH-1 and KMUH-2 genomes as nucleotide queries. The analysis was performed on November 1, 2021. The three most similar sequences that produced significant alignments in the BLAST report were MT479224.1, MW368439.1, and MW514307.1, which featured either one or double deletions that were in a similar position in the spike gene as the deletions of KMUH-1 and KMUH-2. Next, we searched the SARS-COV-2 variants containing spike I68del, spike T76del, spike Q675del, and spike N679del in the GISAID EpiCoV database, and these samples were collected between January 1, 2020 and November 30, 2020. The query results returned included 32 spike I68del strains, 20 spike T76del strains, 59 spike Q675del strains and 70 spike N679del strains. The strains containing spike I68del+spike T76del and the strains containing spike Q675del+spike N679del were manually selected for further analyses. A total of 102 sequences containing Spike 68-76del (32 strains) and/or Spike 675-679del (70 strains) were checked manually for the sequence containing long runs of N, which were not included in the next step of analysis. MT479224.1 was removed at this stage because the same sequence was also deposited in the GISAID EpiCoV database. A panel of 72 sequences containing Spike 68-76del and/or Spike 675-679del retrieved from the GISAID EpiCoV database (70 sequences including KMUH-1 and KMUH-2) and from GenBank (2 sequences) were selected for phylogenetic tree construction. These 72 sequences were aligned using MAFFT v 7.490 (35), and the sequences were trimmed at the 5′ and 3′ ends to give the same size of sequences (29766 nt).

**Figure 2:**
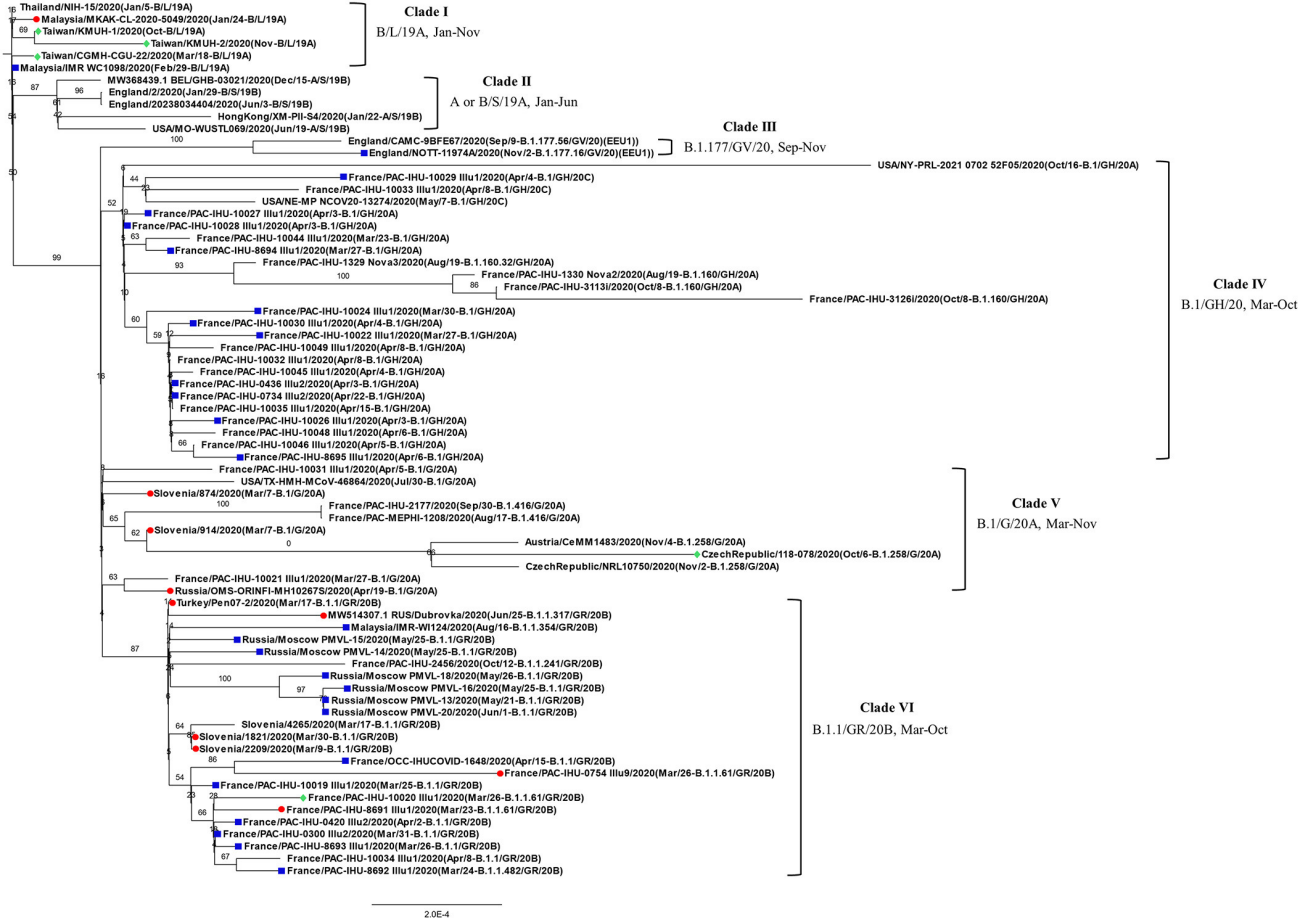
Phylogenetic analysis of 72 closely related SARS-CoV-2 genomes by the maximum likelihood method. The phylogenetic analysis was inferred by using the maximum likelihood method using TIM2+F+I as the best fitted model with the lowest Baysian information criterion (BIC) scores. An original tree was displayed using FigTree v1.4.4 with bootstrap values and a scale bar. The viruses are shown as the virus name (GISAIG) or accession number (GenBank)/sample collection year (date/pangolin_lineage/GISAID_clade/nextstrain_clade). •: Spike 68-76del; ■: Spike 675-679del; ♦ Spike 68-76del+Spike 675-679del double deletion.

**Figure 3:**
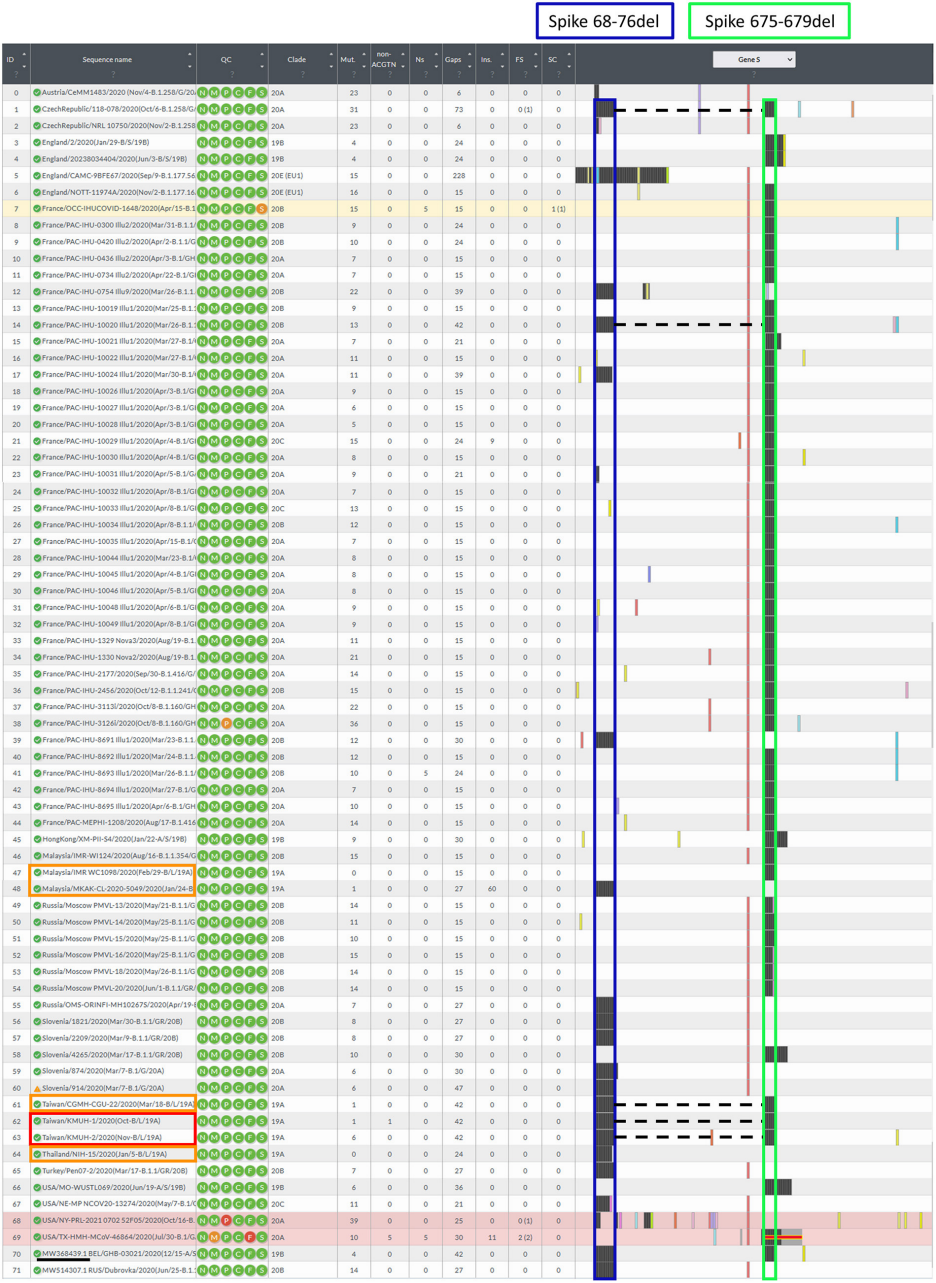
Illustration of the spike gene variants in this study. The spike gene variations in the 72 closely related SARS-CoV-2 genomes were analyzed using Nextclade v1.7.3 using NC_045512.2 SARS-CoV-2 isolate Wuhan-Hu-1/2019 as a reference sequence. Box indicator: Red➔ The two isolates in this study, Orange➔ Phylogenetically closest strains with deletion(s) in the *spike* gene, which were the earliest recorded and deposited in the GISAID EpiCoV database, and the samples were collected between 2020-01-01 and 2020-11-30, Blue➔ Spike 68-76del, Green➔ Spike 675-679del, and the dotted line indicates the double-deletion variants. Other colorful bars indicate amino acid substitutions.

Before construction of the phylogenetic tree of the 72 SARS-COV-2 genomes, the sequences were analyzed by using ModelFinder (36) to find the most appropriate evolutionary model. Maximum likelihood fits of 69 different nucleotide substitution models were computed, and the results suggested TIM2+F+I as the best fitted model with the lowest Baysian information criterion (BIC) scores among the 69 different nucleotide substitution models that were tested. To estimate the ML values, a tree topology was automatically computed. The optimal log likelihood for this computation was −43464.789. There were a total of 29766 positions in the final dataset. Evolutionary analyses were conducted in IQ-TREE 2.1.3 COVID-edition (37). An original tree is shown in Figure 2. The 72 strains were grouped into six phylogenetic clades. The calculation results suggested that the two SARS-CoV-2 isolates were closely related to the strains from the clinical samples that were collected in Thailand (EPI_ISL_444275 Thailand/NIH-15/2020), Malaysia (EPI_ISL_416884 Malaysia/MKAK-CL-2020-5049/2020), and Taiwan (EPI_ISL_444275 Taiwan/CGMH-CGU-22/2020), which belong to clade I. The viruses included in the analysis contained the Spike 68-76del variation, Spike 675-679del variation and Spike 68-76del+Spike 675-679del double-deletion variation are 10, 25, and 6 strains, respectively (Figure 3). Considering that the same deletion event (spike 68-76del and/or spike 675-679del) was relatively rare compared to the same SNVs, the two SARS-CoV-2 isolates were closest to EPI_ISL_416884 Malaysia/MKAK-CL-2020-5049/2020, EPI_ISL_444275 Taiwan/CGMH-CGU-22/2020, and EPI_ISL_430442 Malaysia/IMR WC1098/2020.

## Discussion

In this study, we isolated two SARS-CoV-2 strains from nasopharyngeal swabs from Taiwanese travelers who returned from the United Arab Emirates and the Philippines. Notably, the KMUH-2 strain was isolated by using VREO E6 cell culture from a nasopharyngeal swab collected 7 days post-symptom onset and featuring relatively high Ct values for qRT–PCR. The two isolates had the same double deletion, and a few SNVs across the orf1ab, spike and n genes resulted in coverages of 99.85% and 99.84%, respectively, when compared to the reference sequence. The phylogenetic analysis suggested that the two isolates were closely related to the strains collected in Thailand (EPI_ISL_444275) and Malaysia (EPI_ISL_416884). The earliest documented SARS-CoV-2 strains with the Spike 68-76del variation, Spike 675-679del variation and Spike 68-76del+Spike 675-679del double-deletion variation were EPI_ISL_416884 Malaysia/MKAK-CL-2020-5049/2020(Jan/24-B/L/19A), EPI_ISL_416884 Malaysia/IMR WC1098/2020(Feb/29-B/L/19A) and EPI_ISL_444275 Taiwan/CGMH-CGU-22/2020(Mar/18-B/L/19A) (Figure 3), respectively. Notably, EPI_ISL_4168 84 Malaysia/MKAK-CL-2020-5049/2020 (Jan/24-B/L/19A) retained a 60-nt insertion starting from nt position 194, which was not observed in the two isolates in this study and other strains included in the phylogenetic analysis. Although the phylogenetic analysis suggested that the two isolates were phylogenetically closest to the three spike gene variants listed above, it is possible that the two isolates originated from other variants with either the Spike 68-76del variation or the Spike 675-679del variation due to the nature of this rapidly changing RNA virus. It is also possible that the two isolates evolved from a wild-type (non-deletion) strain since the three variants mentioned above emerged only 2–3 months after SARS-CoV-2 was first isolated.

It has been suggested that the presence of a furin cleavage site around the S1/S2 site (~aa 675-685) in wild-type SARS-CoV-2 is required for viral entry into host cells and potentially enhances its pathogenicity compared to SARS-CoV, which lacks this furin clevage site (40). Recently, Cantuti-Castelvetri et al. suggested that a variant with spike 675-679del (QTQTN) (MW718191.1 human/Finland/FIN-1-VE6-P1/2020) loses its furin cleavage site and infects ACE2- and TMPRSS2-overexpressing HEK-293T cells in an NRP1-independent manner. The loss of its furin cleavage site resulted in the impairment of NRP-1-potentiating ACE-2 infection of host cells (17). In addition, Ramirez et al. reported that a human hepatoma cell clone Huh7.5-adapted-SARS2 accumulated genetic changes, including Spike 68-76del in the N-terminal domain. Huh7.5-adapted SARS-CoV-2 (MZ049598.1 human/DNK/SARS-CoV-2 DK-AHH1 cell culture adapted/2020) was able to infect A549 lung cancer cells, which resulted in a CPE, whereas the original SARS-CoV-2 strain was unable to infect A549 cells. They also found that this spike 68-76del variant is more susceptible to IFN-α2b treatment than the original SARS-CoV-2 strain. Notably, spike 68-76del was not observed in SARS-CoV-2 (MZ049597.1 human/DNK/SARS-CoV-2 DK-AHH1) isolated using VERO E6 cells for virus isolation (41). In our opinion, the self-limited mild symptoms of the two COVID-19 patients infected with KMUH-1 and KMUH-2 with the spike 68-76del+spike 675-679del double-deletion variation in this study might have resulted from the combination of the effects of spike 68-76del and 675-679del variations due to its lower infection rate compared to wild-type virus and increased susceptibility to IFN-α2 induced upon infection. However, the impact of this double deletion in the spike gene on SARS-CoV-2 itself as well as on host cells is largely unknown. The double-deletion variant identified in this study needs to be investigated further to answer this question.

The clinical management and prevention of COVID-19 greatly depend on the timely and accurate identification of people who are infected by SARS-CoV-2. NAATs (e.g., RT–PCR) are performed to detect trace amounts of the genomic sequence of a pathogen even when the patients do not have clinical signs or symptoms. However, RT–PCR can only amplify specific target sequences of SARS-CoV-2 but cannot distinguish genomic fragments from infectious viruses, and it cannot quantify the live virus present in clinical specimens. Several studies have suggested that it is difficult to isolate SARS-CoV-2 from clinical samples with a Ct >30~35 (26, 42–46). Among these studies, Huang et al. reported that the virus was culturable in one out of 38 samples (Ct > 34-35), with the highest Ct = 35.2 targeting the N gene using VERO E6 cells in virus culture (43). La Scola et al. reported that “No culture was obtained from samples with a Ct > 34” targeting the E gene (0/7) using VERO E6 cells in virus culture (44). Singanayagam et al. reported that “virus propagation was successful from five of sixty samples with a Ct > 35” targeting the RdRp gene using VERO E6 cells in virus culture (45). These results suggested that SARS-CoV was quite difficult to cultivate from clinical specimens with a Ct > 35. According to the above studies and our results, infectious SARS-CoV-2 virus particles existed, even with a Ct > 35 in the sample. Basile et al. concluded that SARS-CoV-2 culture may be used as a surrogate marker for infectivity and can determine the deisolation protocols for patients who recover clinically but who are still positive for nucleic detection by PCR (26). Moreover, isolation of live virus plays a crucial role in virology research and in epidemiological studies of SARS-CoV-2 infections. In the current study, SARS-CoV-2 was isolated in one out of the three nasopharyngeal swabs with a Ct > 35 in the three genes analyzed (Table 1). We cultivated the virus with a blind passage on day 3 post inoculation of nasopharyngeal swabs in this study. Sample number 4 did not induce a CPE from day 3 through day 14 post inoculation, while the cells developed CPEs 14 days post-inoculation with blind passage on day 3. It has been reported that no positive viral culture was observed using samples from whom the symptom onset to test (STT) was >8 days (42). This may be the reason why the virus was difficult to cultivate from sample number 4; the STT was 7 days, while the STT of sample number 1 was 1 day. The reason why we did not isolate SARS-CoV-2 from sample numbers 2 and 3 might be that the virus was inactivated during collection and/or processing, although viral RNA was detectable because the presence of the viral genome may not represent a transmissible live virus (47). Another possible reason might be that the antibiotics used in cell culture suppress the growth of viruses (48). In addition to using genetically engineered cells (e.g., ACE2-, TMPRSS2-, and NRP1-overexpressing cells) to facilitate the binding of the virus to the target cells (17), it has been discussed that the method of cultivating viruses may be improved by adjusting other factors, such as by using a prolonged incubation of the culture, and a periodic performance of “blind passages” of the infected cell showed increased infectivity to optimize the detection of low titers and/or slow-growing viruses. The inoculation of cell cultures at 32–34°C instead of 37°C may improve the cultivation of the virus because SARS-CoV-2 spreads mainly through active nasopharyngeal viral shedding (10, 49–51). These alternative methods warrant further investigation to optimize the procedures for the isolation of SARS-COV-2 by cell culture.

**Figure 4:**
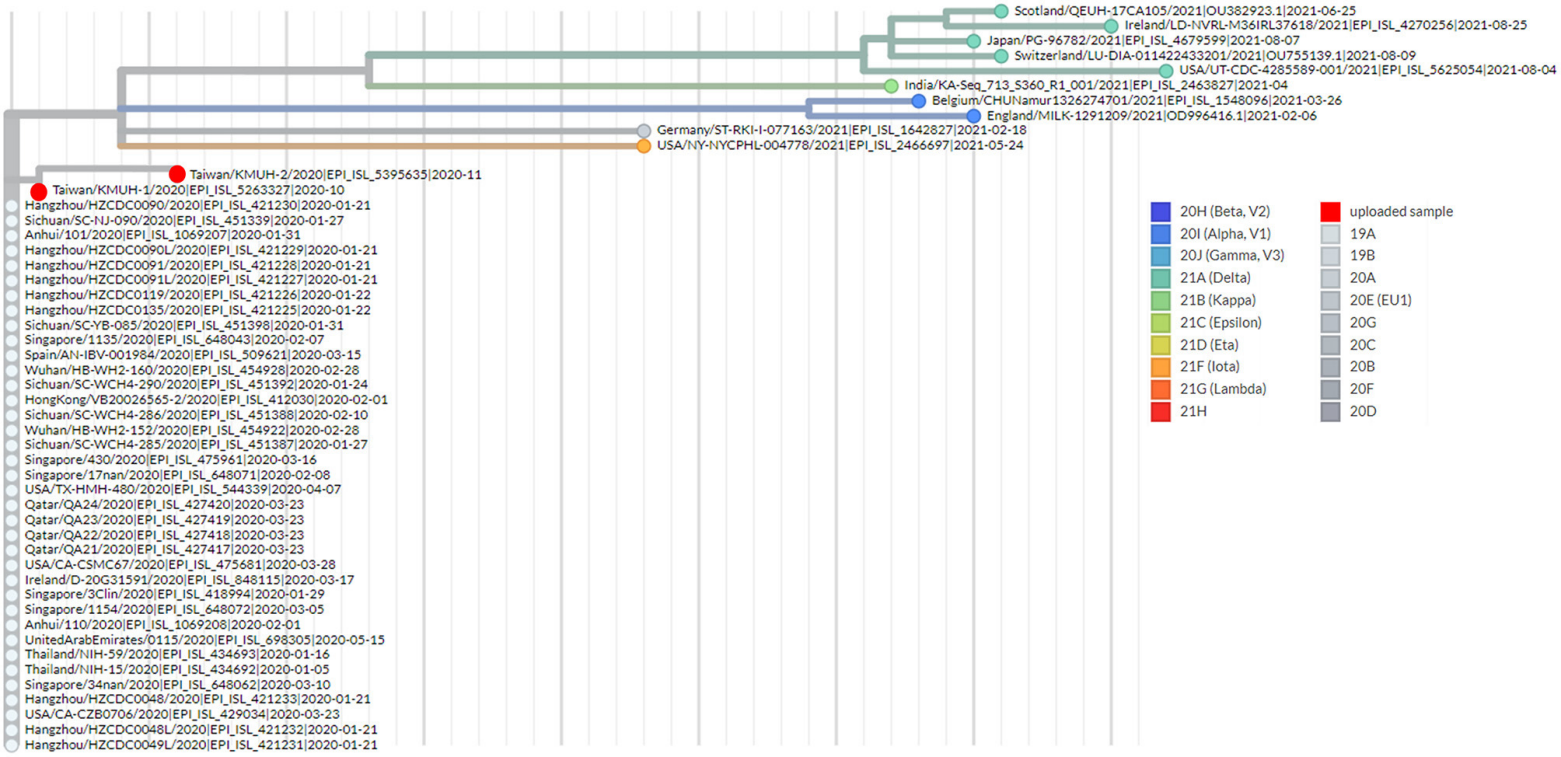
Phylogenetic analysis by using UShER. The phylogenetic tree was generated with UShER using maximum parsimony. UShER enables real-time phylogenetics for the SARS-CoV-2 pandemic using a phylogenetic tree version containing 5,035,953 genomes from GISAID, GenBank, COG-UK, and CNCB (2021-11-12). The phylogenetic tree data were visualized using Auspice v2.32.0 powered by Nextstrain (53). The 50 nearest neighboring GISAID and/or public sequences already in the UShER phylogenetic tree are shown.

To understand the epidemiology of the double-deletion variant identified in this study, we performed a real-time phylogenetic analysis using UShER (Ultrafast Sample placement on Existing tRee) (52) to find the most similar complete and high-coverage SARS-CoV-2 sequences from the GISAID database or from other public sequence databases, such as the NCBI Virus/GenBank, COG-UK and the China National Center for Bioinformation (2021/11/12). The phylogenetic tree containing the two isolates and the 48 nearest neighboring GISAID and/or public sequences is shown in Figure 4. We next performed a BLAST analysis against the coronavirus genomes—NCBI datasets (485,252 complete genomes on 2021/11/12, human host) and GISAID EpiCoV database (5,087,245 viruses on 2021/11/12). One hundred hits and 30 hits are shown on the results page. These three subsets of SARS-CoV-2 sequences were downloaded and validated for the presence of the double-deletion variation by using Nextclade v1.7.3 with the Wuhan-Hu-1/2019 sequence used as the reference sequence. The results were unexpected, and no sequence other than the five entries shown in Figure 3 featured the double-deletion variation in the spike gene (Supplementary Figure 4; Supplementary Table 2). Taken together, these results indicate that the rare double-deletion variant identified in this study has never caused superspreading events within the past 2 years, since there were only five isolates containing spike gene double deletions from January 1, 2020 to November 12, 2021 for which clinical samples were collected in France (1), the Czech Republic (1) and Taiwan (3). It is also likely that most of the people infected with this spike protein double-deletion variant resulted in asymptomatic infection or self-limited mild symptoms who did not receive any specific therapy, so that this variant slipped away from the surveillance. These results raise some questions: 1. Why is this double-deletion variant so rare (one per million in the database on November 12, 2021)? 2. Why did this double-deletion variant not cause superspreading events? 3. What is the difference in the clinical symptoms and outcome of COVID-19 between the patients infected with this double-deletion variant and those infected with the wild-type (WT), the Variants of Concern (VOC) or the Variants of Interest (VOI)? 4. What is the difference in the immune response status of patients infected with this double-deletion variant and those infected with the WT, VOC and VOI? The final question is whether the antibody induced by the spike gene double-deletion variant neutralizes the WT, VOC and VOI SARS-CoV-2. If so, this rare double-deletion variant causing self-limited mild symptoms, at least in this study, may play a role in the development of SARS-CoV-2 vaccines and in the prevention and control of COVID-19 with the oral polio vaccine in mind.

One limitation of this study is that the clinical samples we obtained are rare due to the Taiwanese government's rapid, coordinated, and early response, which was learned from experience during the SARS-CoV outbreak in 2003 (54). Thus, we cannot systematically test the effects of the parameters mentioned above (e.g., blind passage and/or incubation of the cells at 32–34°C instead of 37°C) on the SARS-CoV-2 isolation rate in the samples with a high Ct (e.g., Ct >35). In addition, the Ct value reported in this study might not necessarily be comparable to all of the other studies, since no single procedure for sample collection, PCR reagents, primer sequences, PCR machines, and SARS-CoV-2 genes are standardized between laboratories, not to mention the other parameters such as the cell type and medium conditions used in virus culture, the specimen type and/or the variation of STT. Finally, the results of the phylogenetic analysis in this study only explain the distance between the two isolates and other strains but do not demonstrate the evolutionary history or determine the geographic relationship between them since the traveling history of patients was not disclosed by the SARS-CoV-2 sequence submitters.

## Conclusion

In conclusion, we isolated two SARS-CoV-2 viruses from two nasopharyngeal swabs collected from travelers who returned from the United Arab Emirates and the Philippines by using VERO E6 cell culture. Notably, KMUH-2 was isolated from the samples collected 7 days STT with a Ct > 35 in all three of the genes that were analyzed using qRT–PCR using cell culture with blind passage on day 3, the day CPE was not observed under microscope examination in our lab. Our results indicated that infectious SARS-CoV-2 virus particles existed, even with a Ct > 35 in the sample. Cultivable viruses could provide additional consideration for releasing these patients from quarantine. In addition, we identified a rare double deletion in the spike gene by using bioinformatics analysis, RT–PCR and Sanger sequencing in these two isolates. To our knowledge, this spike 68-76del+spike 675-679del double-deletion variation has never been reported. The impacts of this double deletion in the spike gene on the SARS-CoV-2 virus itself as well as on cultured cells and/or humans are largely unknown. More investigations need to be conducted to answer the question raised above.

## Data Availability Statement

The datasets presented in this study can be found in online repositories. The names of the repository/repositories and accession number(s) can be found at: https://www.ncbi.nlm.nih.gov; OL739246 (KMUH-1) and OL739269 (KMUH-2).

## Ethics Statement

The studies involving human participants were reviewed and approved by Institutional Review Board of KMUH. The patients/participants provided their written informed consent to participate in this study.

## Author Contributions

J-JT, C-HC, and I-WC: resources. J-JT, P-CL, W-LC, J-MC, S-JH, and I-WC: investigation. J-JT, C-HC, P-CL, C-YT, Y-YT, and M-CH: methodology. L-TL, P-CL, C-YT, and Y-YT: data curation. J-JT, P-CL, and C-HC: conceptualization. J-JT, C-HC, W-LC, J-MC, S-JH, and I-WC: supervision. L-TL, J-JT, C-HC, W-LC, J-MC, S-JH, and I-WC: validation. L-TL, J-JT, and P-CL: writing—original draft. L-TL and J-JT: writing—review and editing. All authors contributed to the article and approved the submitted version.

## Funding

This work was supported by the National Health Research Institutes, Taiwan (NHRI-110A1-MRCO-03212101, https://www.nhri.edu.tw/eng to J-JT) and the Ministry of Health and Welfare, Taiwan (MOHW109-TDU-B-212-114006 and MOHW110-TDU-B-212-124006, https://www.mohw.gov.tw/mp-2.html to J-JT). The funders had no role in the study design, data collection and analysis, decision to publish, or preparation of the manuscript.

## Conflict of Interest

The authors declare that the research was conducted in the absence of any commercial or financial relationships that could be construed as a potential conflict of interest.

## Publisher's Note

All claims expressed in this article are solely those of the authors and do not necessarily represent those of their affiliated organizations, or those of the publisher, the editors and the reviewers. Any product that may be evaluated in this article, or claim that may be made by its manufacturer, is not guaranteed or endorsed by the publisher.
