# Supplementary material for: Isolation and Identification of a Rare Spike Gene Double-Deletion SARS-CoV-2 Variant From the Patient With High Cycle Threshold Value
Source: Front Med (Lausanne). 2022 Jan 6;8:822633. doi: 10.3389/fmed.2021.822633 (PMC8770430; doi:10.3389/fmed.2021.822633)
Supplement: Supplementary file 1 [file Table_1.DOC]

**TABLE S1 |** qRT-PCR primers, probe, mixture, machine and cycling conditions.

**A. Primers and probes used in SARS-CoV-2 qRT-PCR**

| E_ Sarbeco_F1 Forward primer | 5’-ACAGGTACGTTAATAGTTAATAGCGT-3’ |
| --- | --- |
| E_Sarbeco_R2 Reverse primer | 5’-ATATTGCAGCAGTACGCACACA-3’ |
| E_Sarbeco_P1 Probe | 5’-FAM-ACACTAGCCATCCTTACTGCGCTTCG-BBQ-3’ |
| RdRp_SARSr-F2 Forward primer | 5’-GTGARATGGTCATGTGTGGCGG-3’ |
| RdRp_SARSr_R1 Reverse primer | 5’-CARATGTTAAASACACTATTAGCATA-3’ |
| RdRp_SARSr-P2 Probe | 5’-FAM-CAGGTGGAACCTCATCAGGAGATGC-BBQ-3’ |
| RdRp_SARSr-P1 Probe | 5’-FAM-CCAGGTGGWACRTCATCMGGTGATGC-BBQ-3’ |
| N_ Sarbeco_F1 Forward primer | 5’-CACATTGGCACCCGCAATC-3’ |
| N _Sarbeco_R1 Reverse primer | 5’-GAGGAACGAGAAGAGGCTTG-3’ |
| N_Sarbeco_P1 Probe | 5’-FAM-ACTTCCTCAAGGAACAACATTGCCA-BBQ-3’ |

**B. qRT-PCR mixture composition**

**LightCycler® Multiplex RNA Virus Master kit (Roche Diagnostics, Mannheim, Germany. Catalog number: 6754155001)**

| E, N gene | reaction µL |
| --- | --- |
| RT Enzyme Solution | 0.1 |
| RT-qPCR Reaction Mix | 4 |
| Water, PCR Grade | 8.4 |
| Forward Primer (10μM) | 1 |
| Reverse Primer (10μM) | 1 |
| Probe (5μM) | 0.5 |
| Template RNA | 5 |
| Total Volume | 20 |

| RdRP gene | reaction µL |
| --- | --- |
| RT Enzyme Solution | 0.1 |
| RT-qPCR Reaction Mix | 4 |
| Water, PCR Grade | 7.3 |
| Forward Primer (10μM) | 1.2 |
| Reverse Primer (10μM) | 1.6 |
| Probe (5μM) | 0.8 |
| Template RNA | 5 |
| Total Volume | 20 |

**C. qRT-PCR cycling conditions**

**PCR thermal cycler Mx3000P (Agilent, USA)**

| Cycles | Temperature | Time |  |
| --- | --- | --- | --- |
| 1x | 50 ^0^C | 10 min | Reverse Transcription |
| 1x | 95 ^0^C | 30 sec |  |
| 45x | 95 ^0^C | 5 sec | Denaturation |
|  | 53 ^0^C | 15 sec | Annealing |
|  | 60 ^0^C | 15 sec | Extension |

**D. Primer sequences, annealing temperature and amplicon length for Validation of Variants by RT-PCR amplification and sanger sequencing**

| **Position** | **Primer Name** | **Primer sequence (5'-3')** | **Annealing (°C**) | **Amplicon (bp)** |
| --- | --- | --- | --- | --- |
| 21532-22220 | Spike-21532-689bp-F | GTGATGTTCTTGTTAACAACTAAACG | 60 | 689 |
|  | Spike-22220-689bp-R | AAACCCTGAGGGAGATCACG |  |  |
| 23366- 24017 | Spike-23366-652bp-F | CAAATACTTCTAACCAGGTTGCT | 60 | 652 |
|  | Spike-24017-652bp-R | TCAATAAATGACCTCTTGCTTGG |  |  |
| 24925-25440 | Spike-24925-652bp-F | GTGTCTGGTAACTGTGATGTTGT | 60 | 652 |
|  | Spike-25440-652bp-R | GCTTCAAAGTTACAGTTCCAATTGTG |  |  |
