# Supplementary material for: Isolation and Identification of a Rare Spike Gene Double-Deletion SARS-CoV-2 Variant From the Patient With High Cycle Threshold Value
Source: Front Med (Lausanne). 2022 Jan 6;8:822633. doi: 10.3389/fmed.2021.822633 (PMC8770430; doi:10.3389/fmed.2021.822633)
Supplement: Supplementary file 2 [file Table_2.DOCX]

**TABLE S2 |** SARS-CoV-2 sequences with either Spike 68-76del or Spike 675-679del variation in GenBank and GISAID database.^a^

|  | seqName | Nextstrain_clade | nt deletions |
| --- | --- | --- | --- |
| Spike 68-76del | MW514307.1 human/RUS/Dubrovka/2020 | 20B | 21764-21790 |
|  | MZ049598.1 human/DNK/SARS-CoV-2 DK-AHH1 cell culture adapted/2020 | 20C | 21764-21790 |
|  | hCoV-19/Italy/CAM-TIGEM-IZSM-COLLI-16185/2021 | 20A | 21764-21790 |
|  | hCoV-19/Italy/CAM-TIGEM-IZSM-COLLI-16069/2021 | 20A | 21764-21790 |
|  | hCoV-19/Italy/CAM-TIGEM-IZSM-COLLI-16084/2021 | 20A | 21764-21790 |
|  | hCoV-19/Italy/CAM-TIGEM-IZSM-COLLI-16076/2021 | 20A | 21764-21790 |
|  | hCoV-19/Italy/CAM-TIGEM-IZSM-COLLI-16188/2021 | 20A | 21764-21790 |
|  | hCoV-19/Italy/CAM-TIGEM-IZSM-COLLI-16191/2021 | 20A | 21764-21790 |
|  | hCoV-19/Italy/CAM-TIGEM-IZSM-COLLI-16190/2021 | 20A | 21764-21790 |
| Spike 675-679del | MZ934691.1 human/FIN/hCoV-19-Finland-1/2020, complete genome | 19A | 23585-23599 |
|  | MW718194.1 human/Finland/FIN-1-VE6-P4/2020, complete genome | 19A | 23585-23599 |
|  | MW718193.1 human/Finland/FIN-1-VE6-P3/2020, complete genome | 19A | 23585-23599 |
|  | MW718192.1 human/Finland/FIN-1-VE6-P2/2020, complete genome | 19A | 23585-23599 |
|  | MW718191.1 human/Finland/FIN-1-VE6-P1/2020, complete genome | 19A | 23585-23599 |
|  | England/NOTT-11974A/2020(Nov/2-B.1.177.16/GV/20E(EU1)) | 20E (EU1) | 23585-23599 |
|  | France/OCC-IHUCOVID-1648/2020(Apr/15-B.1.1/GR/20B) | 20B | 23585-23599 |
|  | France/PAC-IHU-0436 Illu2/2020(Apr/3-B.1/GH/20A) | 20A | 23585-23599 |
|  | France/PAC-IHU-0734 Illu2/2020(Apr/22-B.1/GH/20A) | 20A | 23585-23599 |
|  | France/PAC-IHU-10019 Illu1/2020(Mar/25-B.1.1/GR/20B) | 20B | 23585-23599 |
|  | France/PAC-IHU-10022 Illu1/2020(Mar/27-B.1/GH/20A) | 20A | 23585-23599 |
|  | France/PAC-IHU-10026 Illu1/2020(Apr/3-B.1/GH/20A) | 20A | 23585-23599 |
|  | France/PAC-IHU-10027 Illu1/2020(Apr/3-B.1/GH/20A) | 20A | 23585-23599 |
|  | France/PAC-IHU-10028 Illu1/2020(Apr/3-B.1/GH/20A) | 20A | 23585-23599 |
|  | France/PAC-IHU-10030 Illu1/2020(Apr/4-B.1/GH/20A) | 20A | 23585-23599 |
|  | France/PAC-IHU-10032 Illu1/2020(Apr/8-B.1/GH/20A) | 20A | 23585-23599 |
|  | France/PAC-IHU-10033 Illu1/2020(Apr/8-B.1/GH/20C) | 20C | 23585-23599 |
|  | France/PAC-IHU-10034 Illu1/2020(Apr/8-B.1.1/GR/20B | 20B | 23585-23599 |
|  | France/PAC-IHU-10035 Illu1/2020(Apr/15-B.1/GH/20A) | 20A | 23585-23599 |
|  | France/PAC-IHU-10044 Illu1/2020(Mar/23-B.1/GH/20A) | 20A | 23585-23599 |
|  | France/PAC-IHU-10045 Illu1/2020(Apr/4-B.1/GH/20A | 20A | 23585-23599 |
|  | France/PAC-IHU-10046 Illu1/2020(Apr/5-B.1/GH/20A) | 20A | 23585-23599 |
|  | France/PAC-IHU-10048 Illu1/2020(Apr/6-B.1/GH/20A) | 20A | 23585-23599 |
|  | France/PAC-IHU-10049 Illu1/2020(Apr/8-B.1/GH/20A) | 20A | 23585-23599 |
|  | France/PAC-IHU-1329 Nova3/2020(Aug/19-B.1.160.32/GH/20A) | 20A | 23585-23599 |
|  | France/PAC-IHU-1330 Nova2/2020(Aug/19-B.1.160/GH/20A) | 20A | 23585-23599 |
|  | France/PAC-IHU-2177/2020(Sep/30-B.1.416/G/20A) | 20A | 23585-23599 |
|  | France/PAC-IHU-2456/2020(Oct/12-B.1.1.241/GR/20B) | 20B | 23585-23599 |
|  | France/PAC-IHU-3113i/2020(Oct/8-B.1.160/GH/20A) | 20A | 23585-23599 |
|  | France/PAC-IHU-3126i/2020(Oct/8-B.1.160/GH/20A) | 20A | 23585-23599 |
|  | France/PAC-IHU-8692 Illu1/2020(Mar/24-B.1.1.482/GR/20B) | 20B | 23585-23599 |
|  | France/PAC-IHU-8694 Illu1/2020(Mar/27-B.1/GH/20A) | 20A | 23585-23599 |
|  | France/PAC-IHU-8695 Illu1/2020(Apr/6-B.1/GH/20A | 20A | 23585-23599 |
|  | France/PAC-MEPHI-1208/2020(Aug/17-B.1.416/G/20A) | 20A | 23585-23599 |
|  | Malaysia/IMR-WI124/2020(Aug/16-B.1.1.354/GR/20B) | 20B | 23585-23599 |
|  | Malaysia/IMR WC1098/2020(Feb/29-B/L/19A) | 19A | 23585-23599 |
|  | Russia/Moscow PMVL-13/2020(May/21-B.1.1/GR/20B) | 20B | 23585-23599 |
|  | Russia/Moscow PMVL-14/2020(May/25-B.1.1/GR/20B) | 20B | 23585-23599 |
|  | Russia/Moscow PMVL-15/2020(May/25-B.1.1/GR/20B) | 20B | 23585-23599 |
|  | Russia/Moscow PMVL-16/2020(May/25-B.1.1/GR/20B) | 20B | 23585-23599 |
|  | Russia/Moscow PMVL-18/2020(May/26-B.1.1/GR/20B) | 20B | 23585-23599 |
|  | Russia/Moscow PMVL-20/2020(Jun/1-B.1.1/GR/20B) | 20B | 23585-23599 |
| Spike 68-76del Spike 676-680del | MW368439.1 human/BEL/GHB-03021/2020^b^ | 19B | 21764-21790 23588-23602 |

^a^ Data was retrieved on 2021/11/12

^b^ This sequence entry might be mistaken as Spike 68-76del+Spike 675-679del double-deletion variant in visual graphics FIGURE 3.
