# Supplementary material for: Isolation and Identification of a Rare Spike Gene Double-Deletion SARS-CoV-2 Variant From the Patient With High Cycle Threshold Value
Source: Front Med (Lausanne). 2022 Jan 6;8:822633. doi: 10.3389/fmed.2021.822633 (PMC8770430; doi:10.3389/fmed.2021.822633)
Supplement: Supplementary file 3 [file Table_3.DOCX]

**FIGURE S1 |** Cumulated confirmed COVID-19 cases per million people from January 22 to December 31, 2020. Hannah Ritchie, Edouard Mathieu, Lucas Rodés-Guirao, Cameron Appel, Charlie Giattino, Esteban Ortiz-Ospina, Joe Hasell, Bobbie Macdonald, Diana Beltekian and Max Roser (2020) - "Coronavirus Pandemic (COVID-19)". Published online at OurWorldInData.org. Retrieved from: 'https://ourworldindata.org/coronavirus' [Online Resource]

**FIGURE S2 |** Monthly confirmed COVID-19 cases in Kaohsiung city and in Taiwan in 2020. The data of monthly onfirmed COVID-19 cases, including imported and autochthonous cases, were retrieved from the web-based notifiable diseases surveillance system maintained by the Taiwan CDC. Source of data: https://nidss.cdc.gov.tw/nndss/disease?id=19CoV.

**FIGURE S3 |** Cytopathic effect caused by SAR-CoV-2 from a patient with COVID-19 with a Ct> 35. **(A)** Phase-contrast microscopy of VERO E6 cell monolayers at 3 days postinoculation with control medium (Mock) and sample number 4 (Sample 4-UTM), **(B)** Microscopy of VERO E6 cell monolayers at 14 days postinoculation with control medium (Mock), sample number 4 (Sample 4-UTM) and sample number 4 with blind passage on day 3 (Sample 4-UTM: D3 BP).

**FIGURE S4 |** Validation of the double-deletion variation found in KMUH-1 and KMUH-2 through different pipelines using visual graphics by Nextclade with Wuhan-Hu-1/2019 as a reference sequence. **(A)** The sequences shown in FIGURE 4 (except for KMUH-2 as the query sequence) were aligned with the reference sequence. **(B)** The 30 sequences resulting from GISAID BLAST were aligned with the reference sequence. **(C)** The 100 sequences resulting from NCBI BLAST were aligned with the reference sequence. MT479224.1 TWN/CGMH-CGU-22/2020 was the only sequence that had the same double deletion as KMUH-1 and KMUH-2. However, MT479224.1 TWN/CGMH-CGU-22/2020 is the same sequence as EPI_ISL_444275 Taiwan/CGMH-CGU-22/2020 (Mar/18-B/L/19A). Box indicator: Red🡪 Two isolates in this study, Orange🡪 Phylogenetically closest strains with deletion(s) in spike gene, Blue🡪 Spike 68-76del, Green🡪 Spike 675-679del. Other colorful bars indicate amino acid substitutions.
