# Supplementary figures and images for: Isolation and Identification of a Rare Spike Gene Double-Deletion SARS-CoV-2 Variant From the Patient With High Cycle Threshold Value
Source: Front Med (Lausanne). 2022 Jan 6;8:822633. doi: 10.3389/fmed.2021.822633 (PMC8770430; doi:10.3389/fmed.2021.822633)

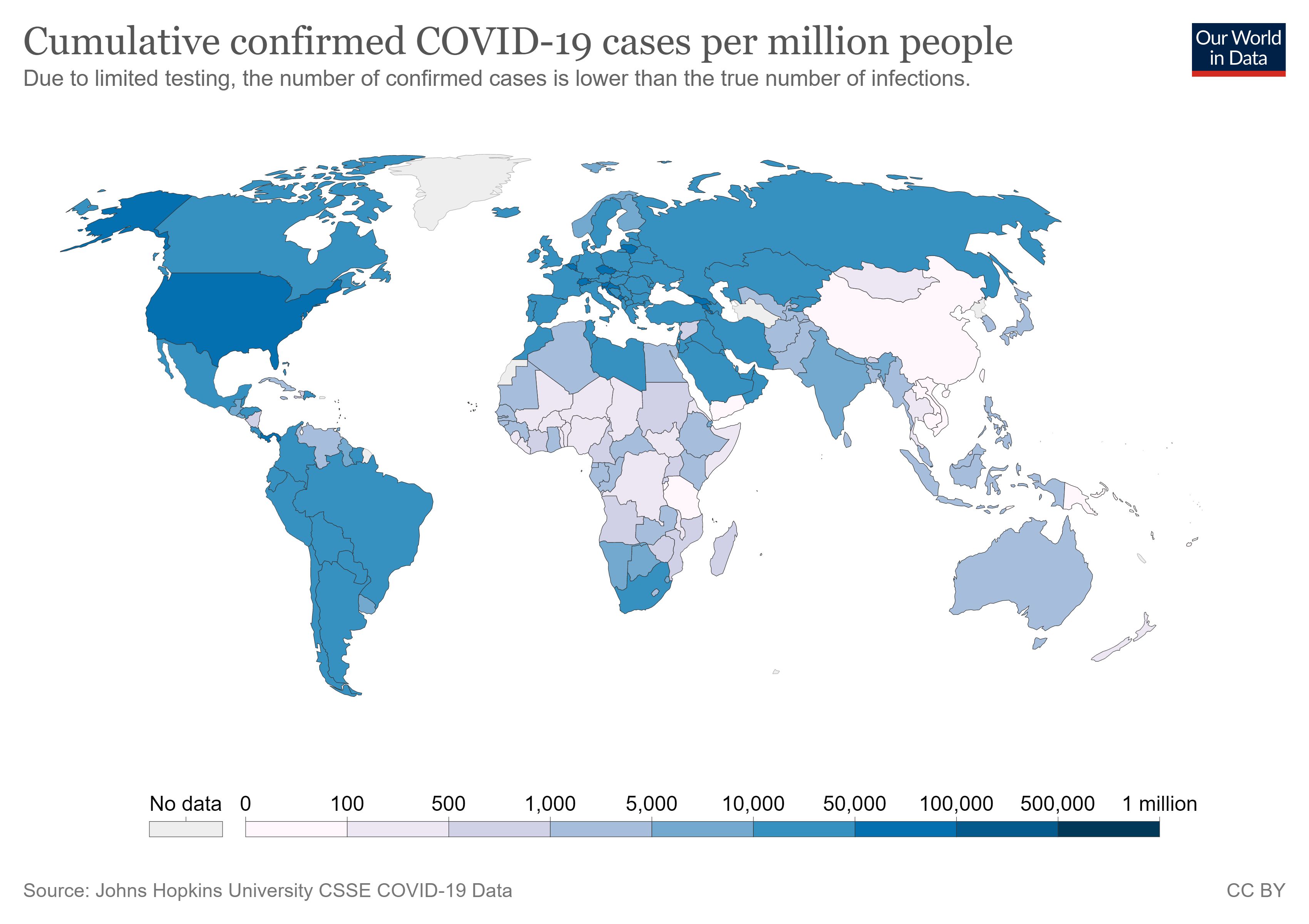

Supplement: Supplementary file 4 [file Image_1.TIF]

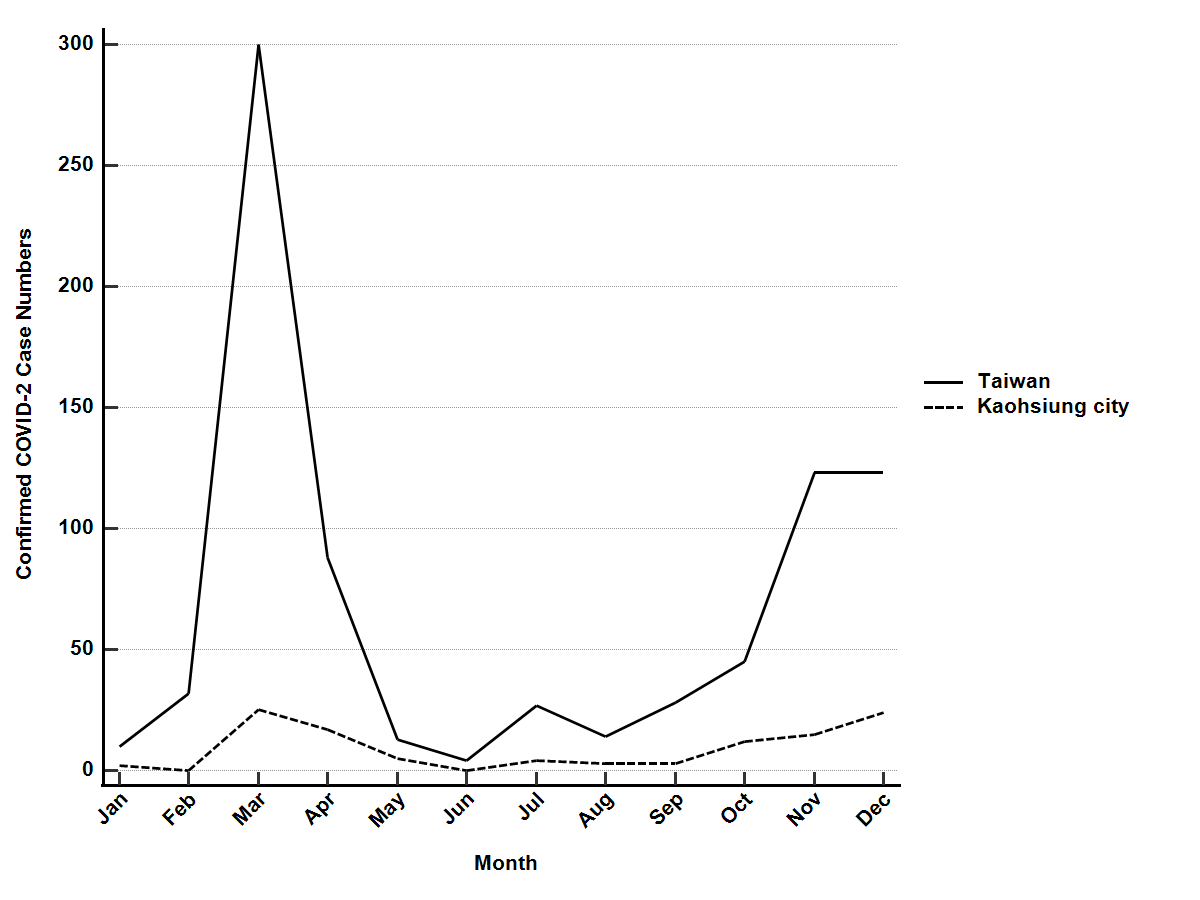

Supplement: Supplementary file 5 [file Image_2.TIF]

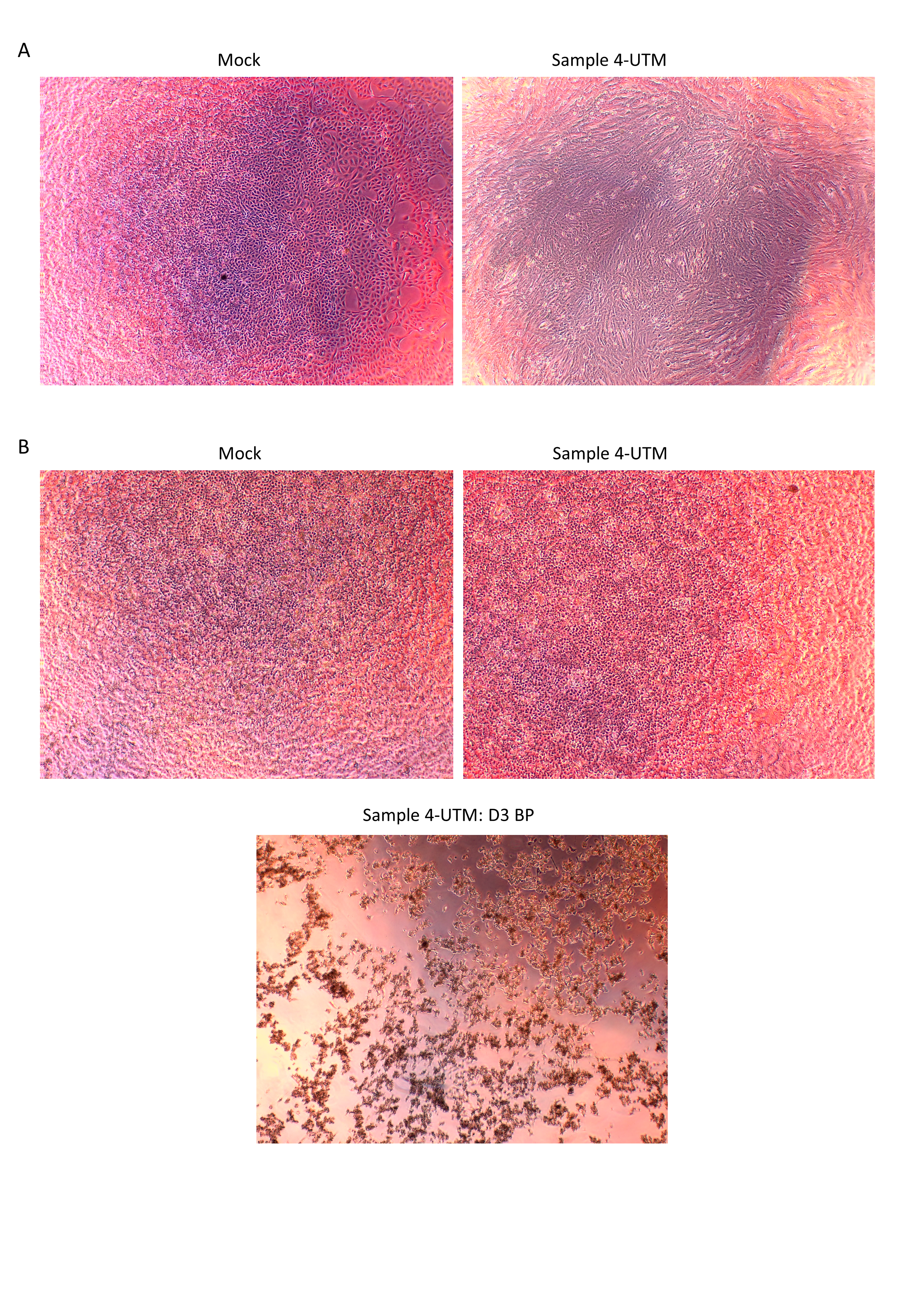

Supplement: Supplementary file 6 [file Image_3.TIF]

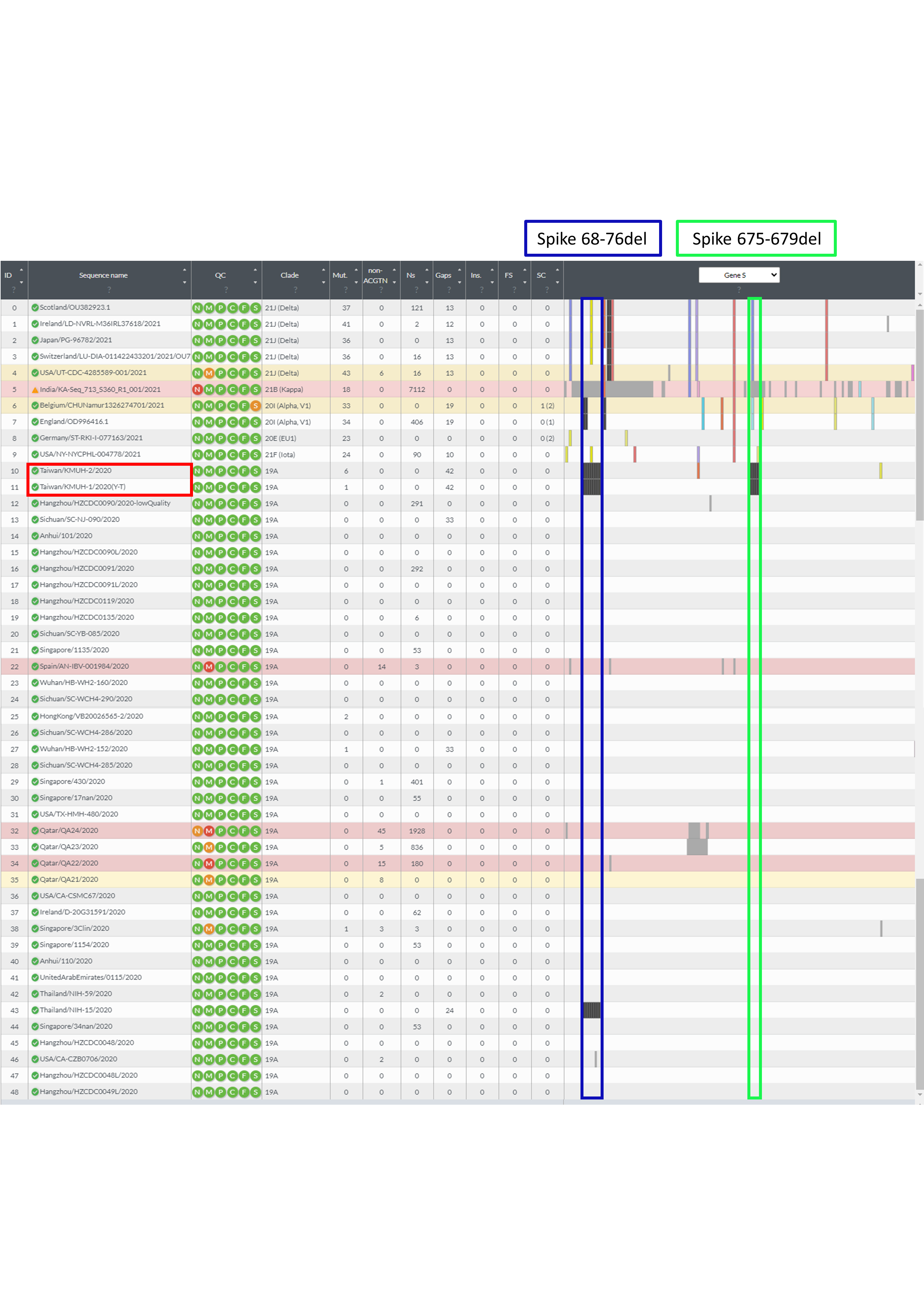

Supplement: Supplementary file 7 [file Image_4.TIF]

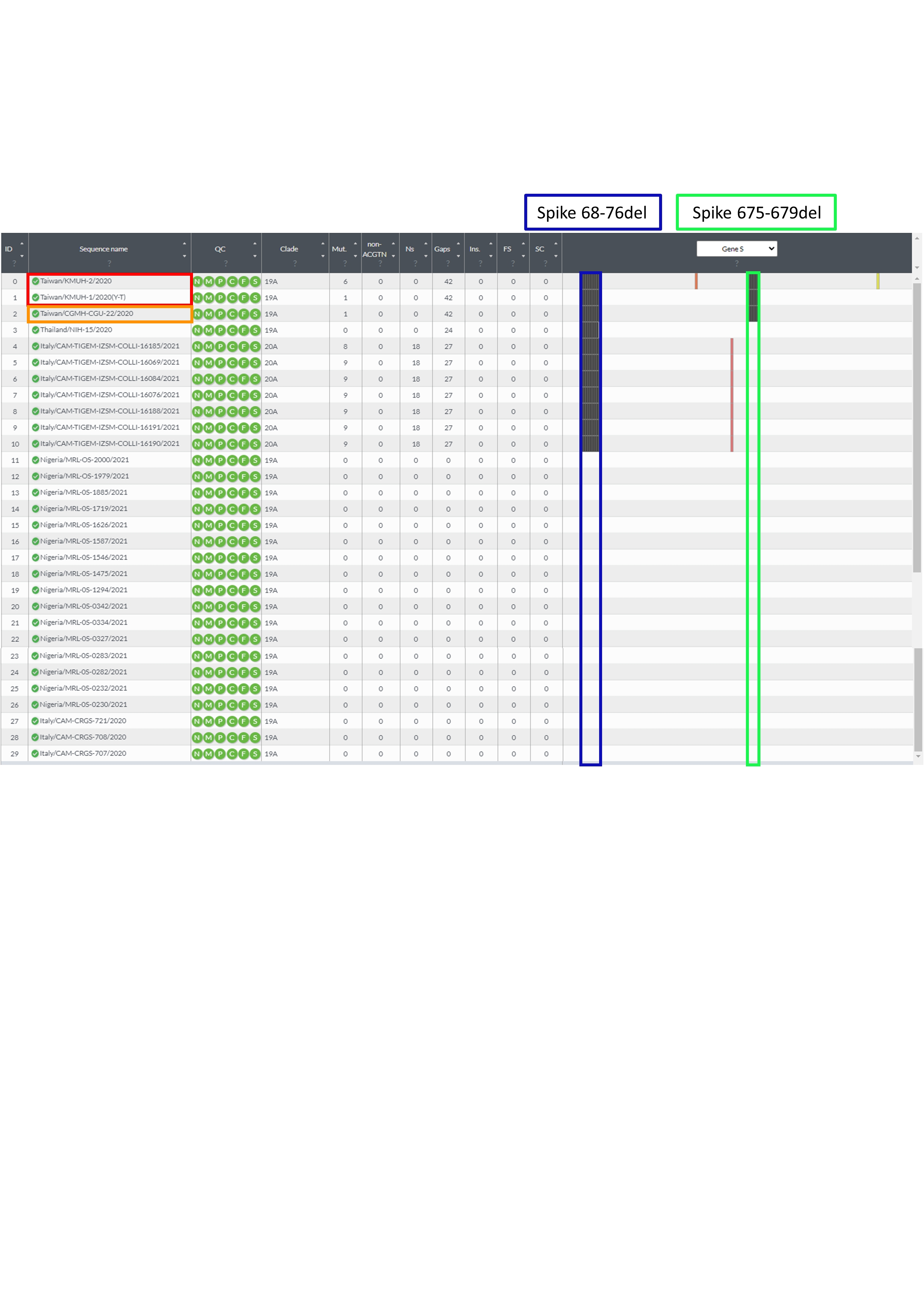

Supplement: Supplementary file 8 [file Image_5.TIF]

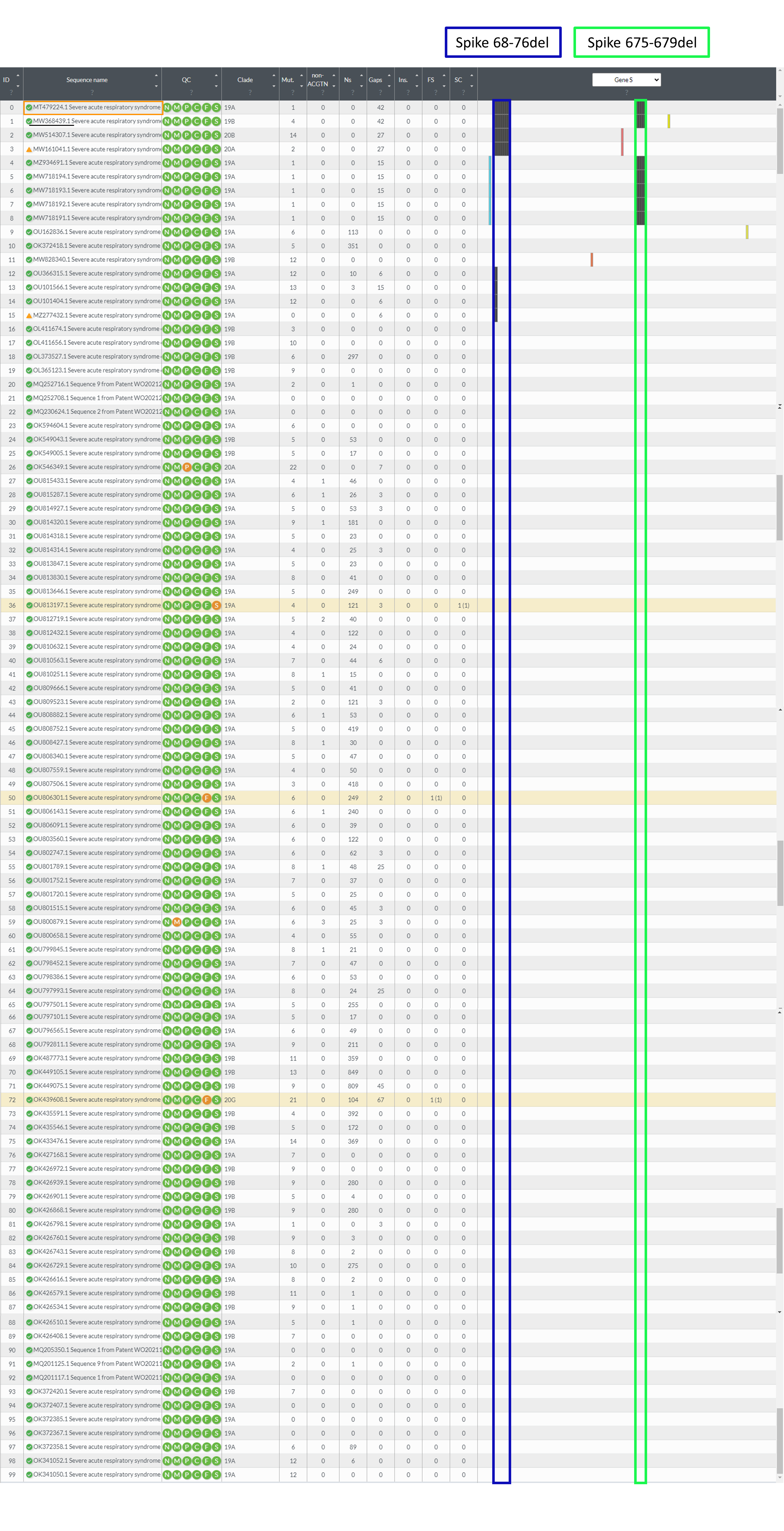

Supplement: Supplementary file 9 [file Image_6.TIF]
